# Supplementary material for: Occurrence and Transport of Isothiazolinone-Type Biocides from Commercial Products to Aquatic Environment and Environmental Risk Assessment
Source: Int J Environ Res Public Health. 2022 Jun 24;19(13):7777. doi: 10.3390/ijerph19137777 (PMC9266048; doi:10.3390/ijerph19137777)
Supplement: Supplementary file 1 [file ijerph-19-07777-s001.zip › ijerph-1776646-supplementary.pdf]

Supplementary Information

Occurrence and transport of isothiazolinone biocide from commercial products to aquatic environment and environmental risk assessment

Iuliana Paun, Florinela Pirvu, Vasile Ion Iancu, Florentina Laura Chiriac*

National Research and Development Institute for Industrial Ecology – ECOIND, Drumul Podu Dambovitei 71-73, Sector 6, 060652, Bucharest, Romania

Table S1. Characteristics and Molecular Properties of the Target Compounds

Compound	Molecular Formula	Molecular Weith	Log Kow
MIT	C_4H_5NOS	115.15	-0.49
CMI	C_4H_4ClNOS	149.60	0.40
BIT	C_7H_5NOS	151.19	1.77
OIT	$C_{11}H_{19}NOS$	213.34	2.45

Table S2. WWTPs and receiving rivers - locations and sample codification

Locality	WWTPs				Receiving Rivers		
	Served population	Average daily flow (m^3/zi)	Sample type	Cod name	Name	Sample type	Cod name
Targoviste (TG)	79600	47606	Influent	IF TG	Ialomita	Upstream	UP TG
			Effluent	EF TG		Downstream	DW TG
			Sewage sludge	SS TG			
Galati (GL)	504000	224640	Influent	IF GL	Siret	Upstream	UP GL
			Effluent	EF GL		Downstream	DW GL
			Sewage sludge	SS GL			
Bucharest (B)	1830000	1028160	Influent	IF B	Dambovita	Upstream	UP B
			Effluent	EF B		Downstream	DW B
			Sewage sludge	SS B			
Iasi (IS)	793500	777600	Influent	IF IS	Bahlui	Upstream	UP IS
			Effluent	EF IS		Downstream	DW IS
			Sewage sludge	SS IS			
Valcea (VL)	110527	88128	Influent	IF VL	Olt	Upstream	UP VL
			Effluent	EF VL		Downstream	DW VL
			Sewage sludge	SS LV			

Table S3. Inter-day and intra-day precision, the method recoveries, limit of detection (LOD) and limit of quantification (LOQ) values determined for commercial products

	RSDr, %	RSD^R, %	Rec, %	LOD	LOQ
MIT	1.12	6.25	89.7	0.13	0.35
BIT	1.37	6.8	88.3	0.13	0.37
OIT	1.75	7.31	91.4	0.06	0.18
CMI	1.44	6.72	88.2	0.24	0.66

Table S4. Inter-day and intra-day precision, the method recoveries, limit of detection (LOD) and limit of quantification (LOQ) values determined for environmental samples

Analytes	wastewater					surface water					sewage sludge				
	RSDr, %	RSD^R, %	Rec, %	LOD, μg/L	LOQ, μg/L	RSDr, %	RSD^R, %	Rec, %	LOD, μg/L	LOQ, μg/L	RSDr, %	RSD^R, %	Rec, %	LOD, μg/g d.w.	LOQ, μg/g d.w.
MIT	2.44	7.02	88.1	0.16	0.44	2.16	5.73	89.2	0.13	0.37	3.01	7.65	87.3	0.004	0.012
BIT	2.65	7.29	87.7	0.15	0.41	2.22	6.51	88.9	0.15	0.42	3.23	8.06	86.5	0.008	0.022
OIT	3.37	8.12	90.3	0.08	0.23	3.10	6.96	92.7	0.06	0.16	3.72	9.74	88.0	0.006	0.018
CMI	3.16	7.33	89.2	0.26	0.74	2.71	6.08	90.3	0.23	0.65	3.53	9.22	87.2	0.012	0.033

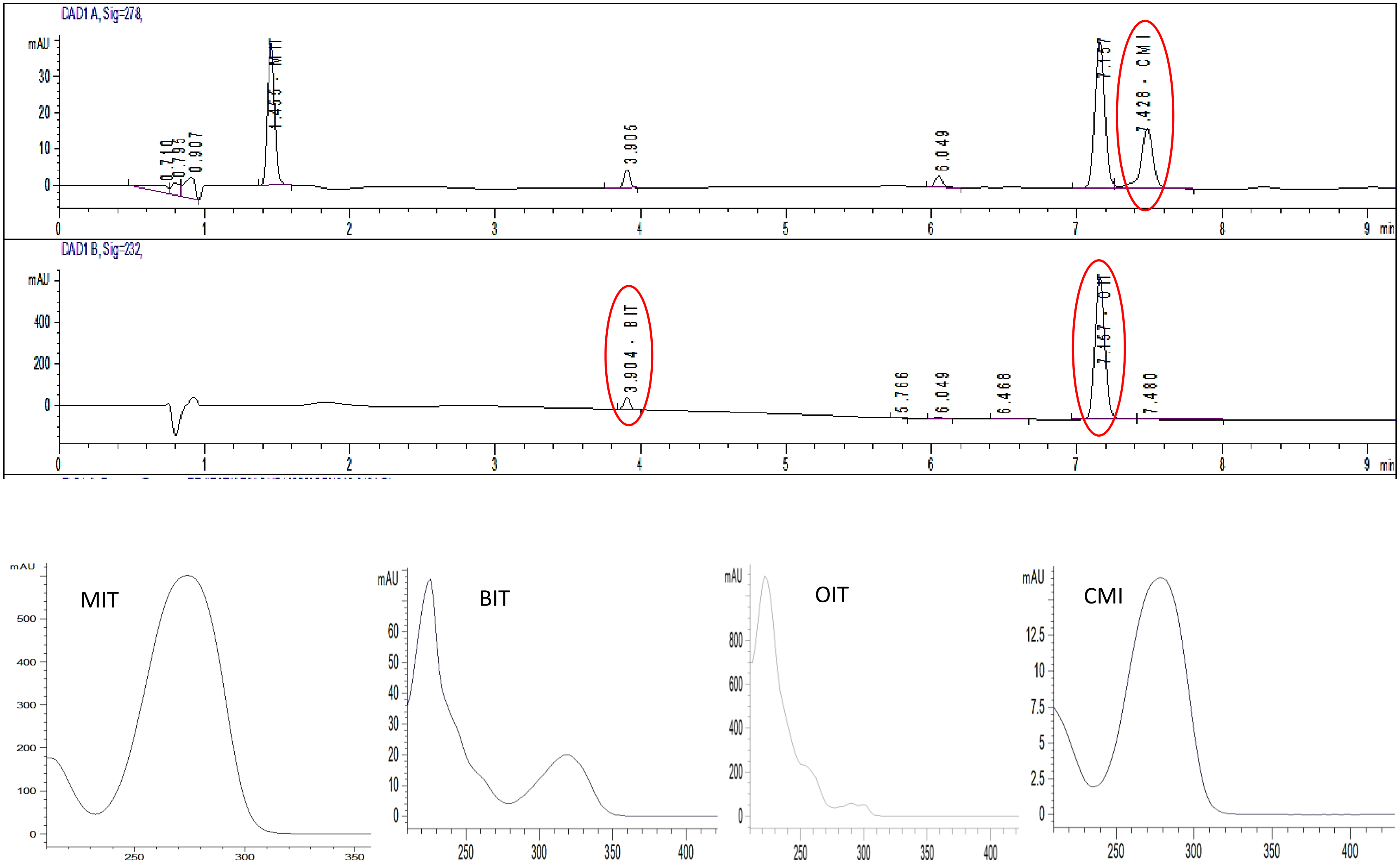

Figure S1. Chromatograms of 5 mg/L biocide standard mixture and their corresponding UV spectrum

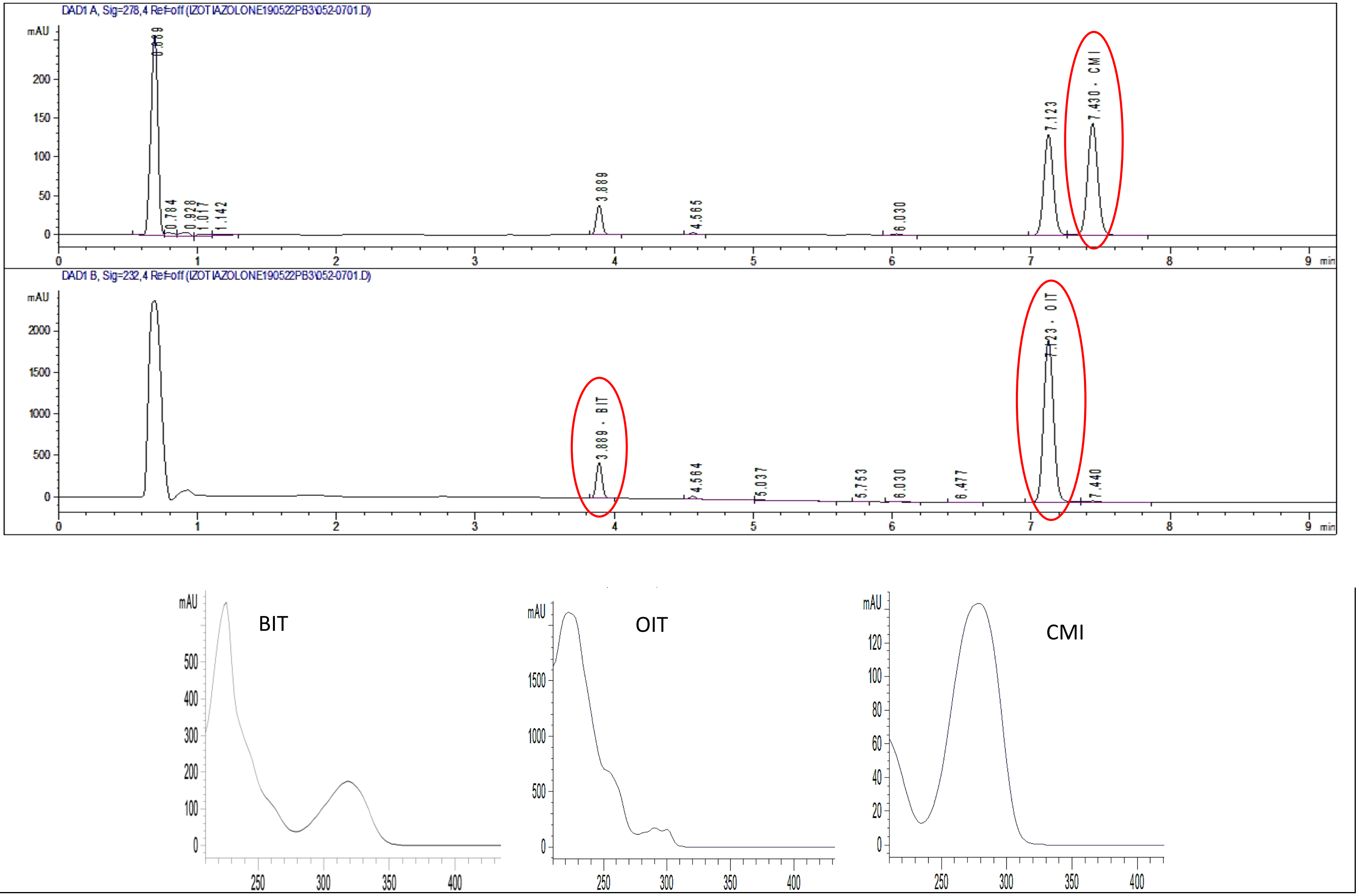

Figure S2. Chromatogram registered for DW VL sample and the corresponding UV spectrum for BIT, OIT and CMI

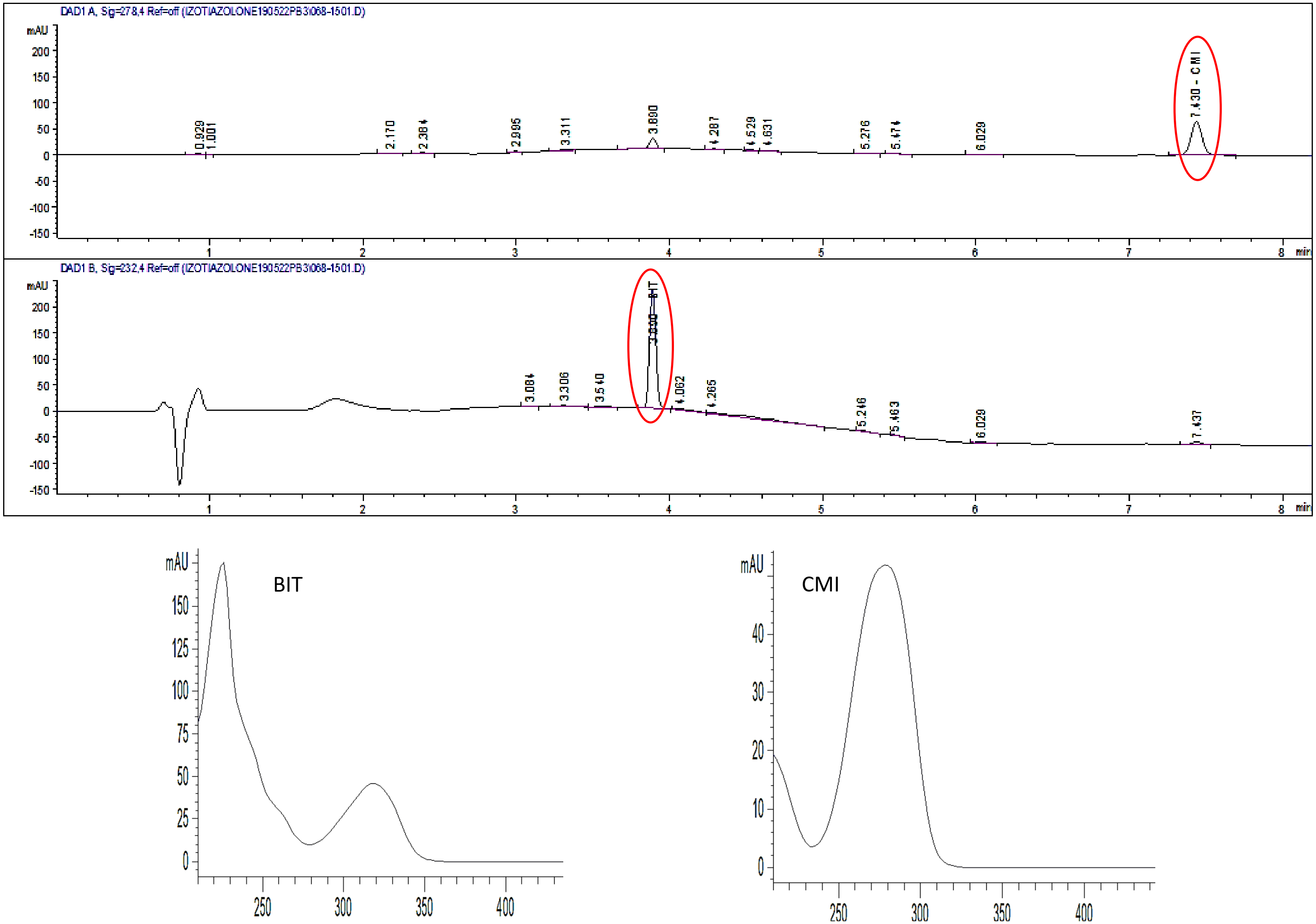

Figure S3. Chromatogram registered for EF VL sample and the corresponding UV spectrum for BIT and CMI

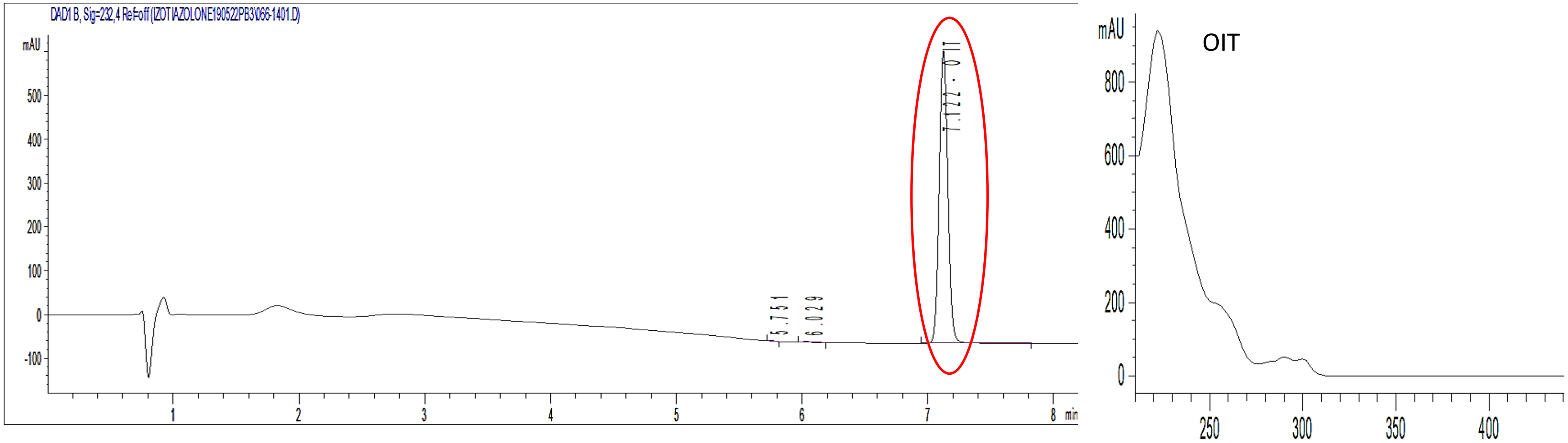

Figure S4. Chromatogram registered for SS GL sample and the corresponding UV spectrum for OIT

Table S5. Summary for the concentration levels (mg/L, mean ± standard derivation, n=3 of 4 biocides in commercial products

Commercial Products	MIT	BIT	OIT	CMI	Commercial Products	MIT	BIT	OIT	CMI
SG 1	6.49±0.07	7.46±0.10	21.1±0.37	3.83±0.06	Sh 1	<LOQ	6.77±0.09	<LOQ	0.66±0.01
SG 2	0.61±0.01	7.21±0.10	74.0±1.30	<LOQ	Sh 2	0.63±0.01	6.26±0.09	12.9±0.23	<LOQ
SG 3	<LOQ	6.60±0.09	67.8±1.19	30.9±0.45	Sh 3	0.63±0.01	7.38±0.10	<LOQ	<LOQ
SG 4	<LOQ	7.71±0.11	<LOQ	<LOQ	Sh 4	<LOQ	3.97±0.05	0.59±0.01	6.75±0.10
LS 1	0.58±0.01	6.37±0.09	65.4±1.14	<LOQ	HC 1	0.51±0.01	8.99±0.12	<LOQ	<LOQ
LS 2	<LOQ	6.49±0.09	66.7±1.17	<LOQ	HC 2	0.64±0.01	9.39±0.13	<LOQ	4.50±0.06
LS 3	<LOQ	7.44±0.10	76.4±1.34	<LOQ	HC 3	0.56±0.01	8.99±0.12	11.7±0.20	<LOQ
LS 4	<LOQ	6.84±0.09	70.3±1.23	2.36±0.03	HC 4	0.50±0.01	9.12±0.12	10.8±0.19	3.20±0.05
DS 1	0.65±0.01	7.26±0.10	<LOQ	1.61±0.02					
DS 2	0.74±0.01	6.64±0.09	<LOQ	<LOQ					
DS 3	<LOQ	7.68±0.11	0.77±0.01	2.20±0.03					
DS 4	<LOQ	6.48±0.09	<LOQ	<LOQ					

Table S6. Summary for the concentration levels (mean ± standard derivation, n=3) of 4 biocides in environmental samples

Water samples	MIT	BIT	OIT	CMI	Sewage sludge samples	MIT	BIT	OIT	CMI
	μg/L					μg/g d.w.			
IF VL	ND	18.2±0.48	2.81±0.09	84.0±2.65	SS VL	ND	0.53±0.02	4.72±0.18	<LOQ
IF IS	ND	<LOQ	ND	34.8±1.10	SS IS	ND	<LOQ	5.19±0.19	<LOQ
IF B	ND	22.9±0.61	0.91±0.03	26.8±0.85	SS B	ND	<LOQ	2.04±0.08	<LOQ
IF GL	ND	36.9±0.98	ND	15.4±0.49	SS GL	ND	<LOQ	5.80±0.22	<LOQ
IF TG	ND	26.2±0.69	ND	32.0±1.01	SS TG	ND	<LOQ	5.55±0.21	2.63±0.09
EF VL	ND	6.58±0.17	ND	18.5±0.59					
EF IS	ND	<LOQ	ND	13.5±0.43					
EF B	ND	5.93±0.16	ND	14.9±0.47					
EF GL	ND	7.57±0.20	ND	5.70±0.18					
EF TG	ND	6.91±0.18	ND	12.5±0.40					
UP VL	ND	47.5±1.06	23.9±0.74	112±3.05					
UP IS	ND	<LOQ	26.6±0.82	105±2.84					
UP B	ND	36.6±0.81	29.1±0.90	45.5±1.23					
UP GL	ND	47.5±1.05	22.3±0.69	29.7±0.81					
UP TG	ND	41.8±0.93	9.19±0.28	2.36±0.06					
DW VL	ND	81.1±1.80	24.3±0.75	144±3.91					
DW IS	ND	40.8±0.91	27.2±0.84	131±3.55					
DW B	ND	47.7±1.06	43.2±1.34	97.4±2.64					
DW GL	ND	55.1±1.22	24.1±0.75	55.3±1.50					
DW TG	ND	61.5±1.37	10.1±0.31	98.7±2.67					

Table S7. Toxicity data: Enf Point, Effect concentration, AF, PNEC

Compound	Specie	Nume	Endpoint	Effect conc (mg/L)	Effect conc (ug/L)	AF	PNEC (ug/L)	Ref
BIT	fish	Oncorhynchus mykiss	LC50	0.167	167	1000	0.167	[23]
	Intervertebrates	Daphnia Magna	EC50	0.097	97	1000	0.097	[23]
	Algae	Selenastrum capricornutum	EC50	0.11	110	1000	0.11	[23]
	Algae	Selenastrum capricornutum	NOEC	0.04	40	100	0.4	[23]
	Bacteria	Activated sludge respiration inhibition	EC50	13	13000	1000	13	[23]
CMI	Algae	Freshwater algal	EC50	0.089	89	1000	0.089	[24]
	Bacteria	Freshwater bacteria	EC50	0.086	86	1000	0.086	[24]
OIT	fish	Oncorhynchus mykiss	LC50	0.047	47	1000	0.047	[25]
	Fish	Pimephales promelas	NOEC	0.0085	8.5	100	0.085	[25]
	Intervertebrates	Daphnia Magna	EC50	0.107	107	1000	0.107	[25]
	Intervertebrates	Daphnia Magna	NOEC	0.074	74	100	0.74	[25]
	Algae	Pseudokirchneriella subcapitata (green algae)	EC50	0.058	58	1000	0.058	[25]
	Algae	Pseudokirchneriella subcapitata (green algae)	NOEC	0.0014	1.4	10	0.14	[25]
	Bacteria	Vibrio fischeri (bioluminescent bacteria)	EC50	0.201	201	1000	0.201	[25]
	Marine intervertebrate	Americamysis bahia (mysid shrimp)	LC50	0.071	71	1000	0.071	[25]
	Marine intervertebrate	Americamysis bahia (mysid shrimp)	NOEC	0.00063	0.63	100	0.0063	[25]
	Marine Mollusc	Crassostrea virginica (oyster)	EC50	0.013	13	10000	0.0013	[25]

Table S8. Risk quotients derived from BIT and OIT

Biocides	MEC UP mean (µg/L)	MEC EF mean (µg/L)	MEC DW mean (µg/L)	MEC UP max (µg/L)	MEC EF max (µg/L)	MEC DW max (µg/L)	Lowest NOEC (mg/L)	AF	PNEC (µg/L)	RQ based on MEC UP mean	RQ based on MEC EF mean	RQ based on MEC DW mean	RQ based on MEC UP max	RQ based on MEC EF max	RQ based on MEC DW max
BIT	35	5	57	48	8	81	0.04	100	0.4	88	13	143	120	20	203
OIT	22	13	105	112	19	144	0.0014	10	0.14	157	93	750	800	136	1029
